# The effects of *Leishmania* RNA virus 2 (LRV2) on the virulence factors of *L. major* and pro-inflammatory biomarkers: an in vitro study on human monocyte cell line (THP-1)

**DOI:** 10.1186/s12866-023-03140-0

**Published:** 2023-12-14

**Authors:** Zahra Mirabedini, Hamed Mirjalali, Elham Kazemirad, Ali Khamesipour, Katayoun Samimirad, Mona Koosha, Reza Saberi, Hanieh Mohammad Rahimi, Mehdi Mohebali, Homa Hajjaran

**Affiliations:** 1 Department of Medical Parasitology and Mycology, School of Public Health, Tehran University of Medical Sciences, Tehran, Iran; 2 Foodborne and Waterborne Diseases Research Center, Research Institute for Gastroenterology and Liver Diseases, Shahid Beheshti University of Medical Sciences, Tehran, Iran; 3 Center for Research and Training in Skin Diseases and Leprosy, Tehran University of Medical Sciences, Tehran, Iran; 4 Department of Virology, School of Public Health, Tehran University of Medical Sciences, Tehran, Iran; 5 Department of Medical Entomology and Vector Control, School of Public Health, Tehran University of Medical Sciences, Tehran, Iran; 6 Toxoplasmosis Research Center, Communicable Disease Institute, Department of Parasitology, School of Medicine, Mazandaran University of Medical Science, Sari, Iran; 7 Center for Research of Endemic Parasites of Iran (CREPI), Tehran University of Medical Sciences, Tehran, Iran

**Keywords:** *Leishmania major*, *Leishmania* RNA virus 2, Virulence factors, Pro-inflammatory biomarkers, *In vitro*

## Abstract

**Background:**

Cutaneous Leishmaniasis (CL) is a parasitic disease with diverse outcomes. Clinical diversity is influenced by various factors such as *Leishmania* species and host genetic background. The role of *Leishmania* RNA virus (LRV), as an endosymbiont, is suggested to not only affect the pathogenesis of *Leishmania*, but also impact host immune responses. This study aimed to investigate the influence of LRV2 on the expression of a number of virulence factors (VFs) of *Leishmania* and pro-inflammatory biomarkers.

**Materials and methods:**

Sample were obtained from CL patients from Golestan province. *Leishmania* species were identified by PCR (LIN 4, 17), and the presence of LRV2 was checked using the semi-nested PCR (RdRp gene). Human monocyte cell line (THP-1) was treated with three isolates of *L*. *major* with LRV2 and one isolate of *L*. *major* without LRV2. The treatments with four isolates were administered for the time points: zero, 12, 24, 36, and 48 h after co-infection. The expression levels of *Leishmania* VFs genes including *GP63*, *HSP83*, and *MPI*, as well as pro-inflammatory biomarkers genes including *NLRP3*, *IL18*, and *IL1β*, were measured using quantitative real-time PCR.

**Results:**

The expression of *GP63*, *HSP83*, and *MPI* revealed up-regulation in LRV2 + isolates compared to LRV2- isolates. The expression of the pro-inflammatory biomarkers including *NLRP3, IL1β*, and *IL18* genes in LRV2- were higher than LRV2 + isolates.

**Conclusion:**

This finding suggests that LRV2 + may have a probable effect on the *Leishmani*a VFs and pro-inflammatory biomarkers in the human macrophage model.

**Supplementary Information:**

The online version contains supplementary material available at 10.1186/s12866-023-03140-0.

## Introduction

Leishmaniasis is a vector-borne disease caused by an intracellular protozoan parasite of the Trypanosomatidae family [1–3]. Leishmaniasis has three clinical forms including cutaneous leishmaniasis (CL), visceral leishmaniasis (VL), and mucocutaneous leishmaniasis (MCL) [4–6].

CL is endemic in tropical and subtropical countries, with a global incidence ranging from 0.7 to 1 million new cases per year [7]. Iran is one of the endemic areas for CL and *L. major*, and *L. tropica* are responsible for approximately 80% and 20% of cases, respectively [4, 6, 8]. Clinical manifestations of CL are mostly limited to skin ulcers, however, atypical forms including disseminated, mucosal, and visceral involvements are also reported [9–11]. The severity of the disease seems to be multifactorial, depending on the host immune responses, *Leishmania* species, and sandfly factors [12, 13].

Recent evidence has highlighted the role of viruses as endosymbionts in the pathogenicity of certain protozoa [14–17]. *Leishmania* RNA virus (LRV) was firstly identified in *L. guyanensis* by Tarr et al. [18]. Based on the complete nucleotide sequence, LRVs are classified into two types: LRV1 (New World) and LRV2 (Old World), with less than 40% similarity in their genomes [19, 20]. The presence of LRV2 in Iran was mostly confirmed in *L. major*, and rarely in *L*. *infantum* and *L. tropica* [21–23]. However, the role of LRV in treatment failure, pathogenesis of *Leishmania* species, and immune responses have been investigated [24–26].

*Leishmania* virulence factors (VFs) play a crucial role in pathogenesis of the parasite by influencing the host’s immune responses [12, 27, 28]. This study examines the most important pathogenesis factors, contributing to the parasite pathogenesis and cytokine regulation.

Heat-shock proteins (HSP), glycoprotein phosphatase (GP63), and mannose phosphate isomerase (MPI) are the most important pathogenesis factors, which play crucial roles in the maturation of *Leishmania* spp., macrophage activation, immune modulation and growth of the parasite, respectively [29–31]. HSPs or stress proteins are highly evolutionarily conserved proteins that play important roles in vital activities of *Leishmania*, such as protection against stress and trivalent antimonials (HSP23), and the maintenance of the cell (HSP90) [16]. HSP90 (HSP83 homolog) is also considered as a viral protein in maturation of the parasite [29].

GP63, a prominent surface protein belonging to the metzincin class, is commonly expressed on the surface of *Leishmania* parasites. It is recognized as the primary membrane surface protein in these parasites. This activity of GP63 is related to the protection of *Leishmania* parasites against phagolysosomes of macrophages in hosts and digestive enzymes in the vector’s midgut [31]. Additionally, GP63 is one of the main factors, which is activated during macrophage infection that modulates immune responses [31, 42].

MPI is an enzyme playing a crucial role in the reversible conversion of fructose-6-phosphate and mannose-6-phosphate, which are essential for the biosynthesis of various glycoconjugates. The absence of MPI has been linked to prolonged growth time in *Leishmania* spp [14]. Additionally, *Leishmania* species produce significant amounts of mannose-containing glycolipids and glycoproteins, which contribute to the virulence factors of *Leishmania* spp [16].

Interleukin (IL) IL-1β and IL-18 are important pro-inflammatory cytokines during innate immune responses to leishmaniasis, which are mediated by activation of NOD-like receptors (NLRs) [32, 33]. The role of NLRP3 in leishmaniasis seems to be like a double-edged sword. Although NLRP3 is thought to be protective against leishmaniasis, there is evidence suggesting a synergistic role of this inflammasome in the pathogenesis of the parasite [34].

While several studies have investigated the role of LRV1 in the pathogenesis of *Leishmania* spp., there is few data on the effects of LRV2 in the pathogenesis of Old World *Leishmania* species [17, 35]. This study aimed to investigate the effects of a number of *L*. *major* (three LRV2 + and one LRV2-) isolates, collected from CL patients, on the expression of VFs (GP63, HSP83, and MPI) in *Leishmania* isolates, and pro-inflammatory biomarkers (NLRP3, IL18, and IL1β) on human monocyte cell line (THP-1).

## Materials and methods

### Sample collection and cultivation

*Leishmania* isolates were collected from CL patients whom were referred to the referral health centers in Golestan province, during December 2021 and May 2022. These patients were diagnosed based on the clinical characteristics and parasitology methods (including microscopic and culture detection). For parasitology diagnosis, suspected lesions to CL were scraped by using a sterile scalpel, and the exudate materials were stained with Giemsa, and microscopically checked. The scrapped materials were initially cultured on a two phasic medium containing Novy-MacNeal-Nicolle (NNN) medium and RPMI-1640 medium (Gibco, Germany) supplemented by 10% fetal bovine serum (FBS) (Gibco, Germany), with penicillin (100 U/mL) and streptomycin (100 µg/mL) (Sigma-Aldrich, St. Louis, USA). The culture media were incubated at 25˚C. After 6–8 days, the promastigotes were sub-cultured and incubated at 25 °C in RPMI-1640 medium, supplemented with 10% FBS and 1% penicillin/streptomycin, for 5 days [23].

#### *Leishmania* species identification

DNA was extracted according to the protocol of the commercial kit (Bioneer Company, Korea). To carry out the PCR, we used the primers Forward (LIN4, 5’ GGGGTTGGTGTAAAATAGGG 3’) and Reverse (LIN17, 5’ TTTGAACGGGATTTCTG 3’) to amplify identical 680 to 720-bp fragments of the kinetoplast (kDNA) gene in *Leishmania* isolates [36]. All the procedures were monitored by standard reference isolates of *L*. *major* (Acc. no. JN860745) and *L. tropica* (Acc. no. EF653267).

#### *Leishmania* RNA virus detection

### RNA extraction and cDNA synthesis

Total RNA was extracted from 1 × 10^6^ promastigotes according to the manufacturer’s protocol (YTZ, Favorgen, Taiwan). The purity of the extracted RNA was evaluated through agarose gel electrophoresis, based on the appearance of the specific bands. Additionally, the concentration of RNA was determined using a NanoDrop spectrophotometer at 260 nm (Thermo Scientific™ NanoDrop™ One Microvolume UV–Vis) (Suppl Fig. 1). The complementary DNA (cDNA) was synthesized from 100 ng of total RNA YTA kit (Favorgen, Taiwan) following the manufacturer’s protocol [17]. The amplified cDNA was stored at -20 °C till to be used for semi-nested PCR.

### Semi-nested PCR

The initial PCR using an outer forward primer LRV F1 (5’ TGTAACCCACATAAACAGTGTGC 3’) and reverse primer LRV R (5’ATTTCATCCAGCTTGACTGGG 3’) was performed to amplify a 526-bp external partial sequence of the *RdRp* gene. The semi-nested PCR was performed on the primary PCR products. A pair of primers, forward primer LRV F2 (5’ AGGACAATCCAATAGGTCGTGT 3’) and reverse primer LRV R (5’ATTTCATCCAGCTTGACTGGG 3’) were used to amplify a 315-bp product of the *RdRp* gene of LRV2. The PCR program for two steps consisted of 35 cycles of 94 °C for 35 s, 60 °C for 35 s, and 72 °C for 1 min. The final extension of the strands consisted of 72 °C for 4 min. The PCR products were analyzed by electrophoresis on a 1.5% agarose gel stained with SYBR safe gel stain (Thermo Fisher Scientific, USA) next to the 100 bp DNA marker (Fermentas, Life Sciences) [17, 37].

### In vitro assays

#### Macrophage differentiation

THP-1 cells were cultured in 25 cm^2^ culture flasks (SPL Life Science Co, Korea) in a complete medium containing RPMI 1640 with 25 mM HEPES, supplemented with 10% FBS and 1% penicillin (100 U/mL) and streptomycin (100 µg/mL) (Sigma-Aldrich, St. Louis, USA). The cells were incubated at 37 °C, with 5% CO_2_. The culture medium within the flasks was changed every 2–3 days. To differentiate THP-1 monocyte to macrophage, 5 × 10^5^ cells/mL were transferred to a 6-well cell culture plate (SPL Life Sciences, Korea) containing RPMI-1640, supplemented with 50 ng/mL phorbol myristate acetate (PMA) (Santa Cruz Biotechnology). The cells were incubated at 37 °C and 5% CO_2_ for 48 h. Differentiated cells were identified by the presence of pseudopodia and adherence to the bottom of the wells, while non-adherent undifferentiated monocytes were washed away with RPMI 1640 media [38].

#### Leishmania culture

Promastigotes of four *L. major* isolates (three LRV2 + and one LRV2-) were sub-cultured in RPMI-1640 with 10% FBS and 1% penicillin-streptomycin every 5–6 days and kept at 25 °C.

### Macrophage Infection

Prior to co-incubation, promastigotes of each *Leishmania* isolate were centrifuged at 2500 rpm for 7 min and the cell pellet was re-suspended with fresh RPMI 1640 medium with 10% FBS. THP-1 macrophages were infected with each *Leishmania* promastigotes isolate with a multiplicity of infection (MOI) = 3 and were incubated at 37 °C with 5% CO_2_. The expression analyzes of target genes were performed at zero (6 h after initial infection), 12, 24, 36, and 48 h after co-infection. All experiments were performed in duplicate.

### RNA extraction and cDNA synthesis

Total RNA was extracted as described by the manufacturer (YTA, favorgen, Taiwan). The zero time-point was described as 6 h after the initial co-infection. The purity of the extracted RNA was assessed by agarose gel electrophoresis (based on the appearance of the specific bands on the gel) and the concentration of RNA was assessed using a NanoDrop spectrophotometer at 260 nm (Thermo Scientific™ NanoDrop™ One Microvolume UV-Vis). The cDNA was synthesized from 500 ng of total RNA using Superscript II Reverse Transcriptase “cDNA synthesis kit” (SMOBIO) following the manufacturer’s instructions. The amplified cDNA was stored at -20 °C till used for real-time quantitative PCR.

### Quantification real-time PCR

Transcriptional analysis of the VFs genes (*GP63, HSP83*, and *MPI*) and pro-inflammatory biomarkers (*NLRP3*, *IL18*, and *IL1β*) was carried out compared to *alpha-tubulin* (*ALT*) and *Beta-Actin (Β-ACT)* genes, as housekeeping genes for *Leishmania* and pro-inflammatory biomarkers, respectively (Table 1).

**Table 1:** Primer sequences for parasite VF genes and pro-inflammatory biomarkers

Genes	Primer name	Sequence 5’-3’	Length (mer)	Tm	CG (%)	Amplification size(bp)	Refs
*Alpha tubulin*	ALT-F	CAGGTGGTTGTCGTCTCTGAC	20	60.04	60	119	(17)
	ALT-R	TAGCTCGTCAGCACGAAGTG	20	60.11	55		
*GP63*	GP63-F	ATCTGTGGCGACTTCAAGGT	20	59.31	50	136	
	GP63-R	CAGAGAACGTCTGGCAGGTC	20	60.39	60		
*MPI*	MPI-F	AGTGCCCTACCTGCTGAAGA	20	59.87	55	138	
	MPI-R	ATGAGCTCTGGCTTGTGGTT	20	59.31	50		
*HSP-83*	HSP83-F	ACGAAGCACTTCTCTGTGGAG	21	59.66	53.38	108	
	HSP83-R	GATGTTGTTGCGCTTCTTGTT	21	60.31	42.38		
*Β-actin*	B-act-F	ATGTGGCCGAGGACTTTGATT	21	60.00	47.62	107	(38)
	B-act-R	AGTGGCCGAGGACTTTGATT	21	59.99	52.38		
*NLRP3*	NLRP3-F	AAGGAAGTGGACTGCGAGA	19	58.25	52.63	127	
	NLRP3-R	TCAAACGACTCCCTGGAAC	19	57.00	52.63		
*IL18*	IL18-F	ATCGGCCTCTATTTGAAGATATGACT	26	59.79	38.46	100	
	IL18-R	CCTCTAGGCTGGCTATCTTTATACATACT	29	61.46	41.38		
*IL1β*	IL1β-F	CAGGGACAGGATATGGAGCAAC	22	60.49	54.55	133	
	IL1β-R	CATCTTTCAACACGCAGGACAG	22	60.10	50		

A real-time PCR was performed in a 15-µl reaction containing: 0.5 µl forward, 0.5 µl reverse primers 7.5 µL 2X SYBR green master mix (Ampliqon, Denmark), 5.5 µl distilled water, and 1 µl cDNA from baseline pure culture or post-macrophage co-infection at zero, 12, 24, 36 and 48 h. The reaction was programmed with the following details: holding stage: at 90 °C/3 min, cycling stage: 45 cycles/15 sec at 95 °C and at 60 °C/35 sec, and melt curve stage: at 95 °C/15 sec, at 60 °C/60 sec and then at 95 °C/15 sec. Results were analyzed using the relative expression software tool (REST; https://www.gene-quantification.de/rest.html). The relative expression value of each gene was determined based on the threshold cycle (Ct) value of the target genes, calculated by normalization with *ALT* and *Β-ACTIN* constitutive gene Ct values. All experiments were duplicated and data are reported as the mean ± SD (standard deviations). The level of accepted statistically significance was 95% and *P*-value < 0.05.

## Results

### *Leishmania* characterization and LRV2 detection

In this study, four *Leishmania* isolates were selected from human CL patients based on the study’s objectives. All four isolates were identified as *L. major* (Suppl Fig. 2). Among these, one isolate (S1-) was LRV2 negative, while three isolates (S2+, S3+, and S4+) were positive for LRV2 using semi-nested PCR (Table 2). These isolates were further utilized for the analysis of VFs genes and pro-inflammatory biomarkers expression using RT-qPCR methods.

**Table 2:** Characteristics of isolates from CL patients

Isolate code	Source of isolate	Nomenclature codes	*Leishmania* species	LRV2 status	Number of lesions	Clinical phenotype
S1-	Clinical	MHOM/IR/21/Leish.z.1	* L. major*	LRV2-	1	Non-sever
S2+	Clinical	MHOM/IR/21/Leish.z.2	* L. major*	LRV2+ (ACC number: OR493488)	7	Non-sever
S3+	Clinical	MHOM/IR/21/Leish.z.3	* L. major*	LRV2+ (ACC number: OR493489)	2	Non-sever
S4+	Clinical	MHOM/IR/21/Leish.z.4	* L. major*	LRV2+ (ACC number: OR493490)	1	Non-sever

### Virulence factors expression

#### GP63

**Fig. 1:**
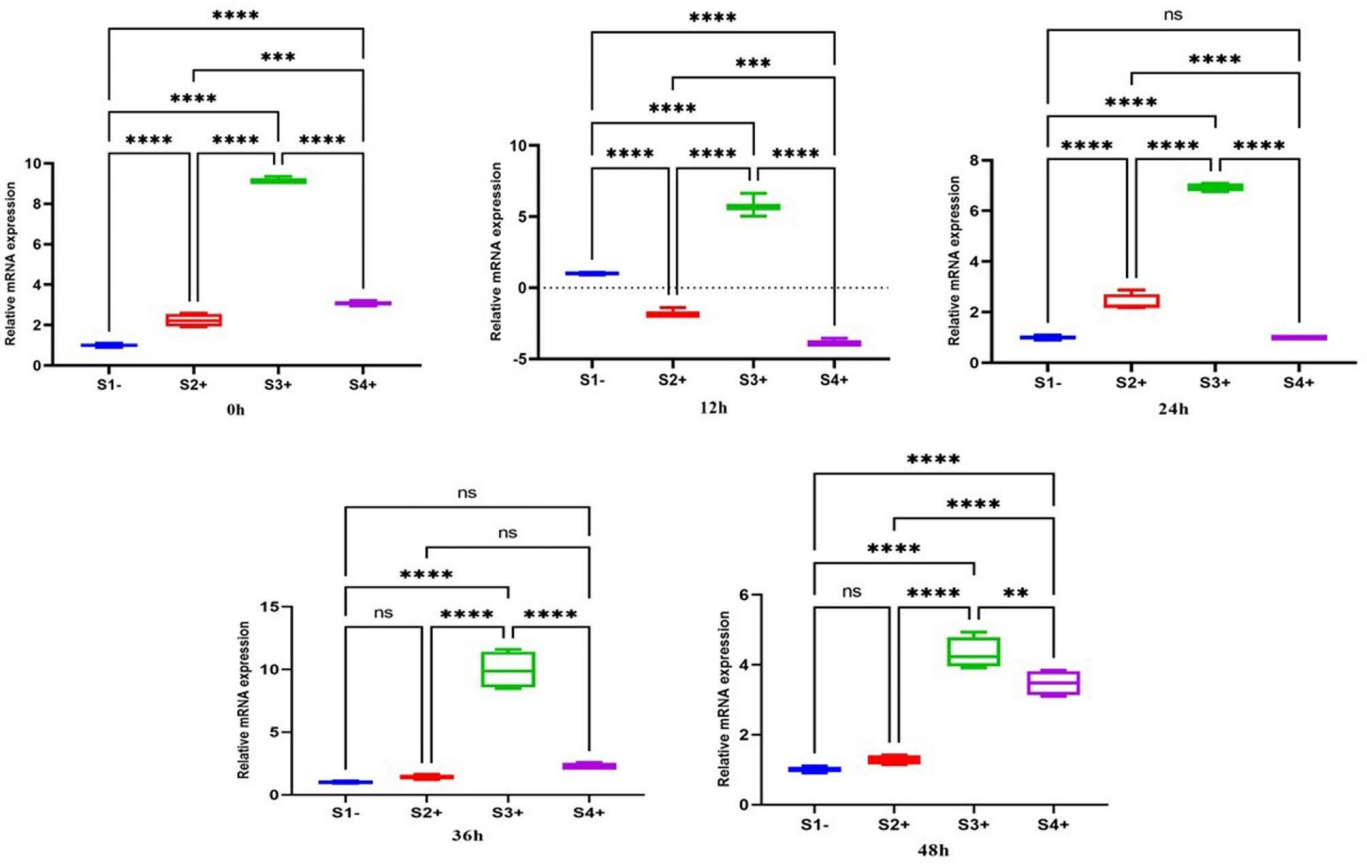
Gene expression of the *GP63* gene in three LRV2 + isolates (S2+, S3+, and S4+) compared to LRV2- isolate (S1-) at different times after co-infection. Data analysis was done using two-way ANOVA for repeated measurements followed by the Tukey test. Bars represent mean ± SD. * Symbol represents the meaningful difference between groups. (*P ≤ 0.05, **P ≤ 0.01, ***P ≤ 0.001, ****P ≤ 0.0001)

The real-time PCR results revealed significant changes in the expression of the *GP63* gene for three LRV2 + isolates (S2+, S3 + and S4+) at the initial infection time of zero, compared to the S1- (LRV-). An upregulation was observed in S2+ (2.23; *P*-value = 0.0001), S3+ (9.21; *P*-value = 0.0001), and S4+ (3; *P*-value = 0.0001). Similarly, the expression of the *GP63* gene was downregulated in S2+ (-2.3; *P*-value = 0.0001), S4+ (-3.7; *P*-value = 0.0001), but showed upregulation in S3+ (6; *P*-value = 0.0001) at 12 h. At the time points of 24, 36, and 48 h, all isolates were upregulated, with the highest gene expressions for *GP63* gene in S3 + at 24 h (6.9; *P*-value = 0.0001), 36 h (9.9; *P*-value = 0.0001), and 48 h (4.3; *P*-value = 0.0001) (Fig. 1).

#### HSP83

**Fig. 2:**
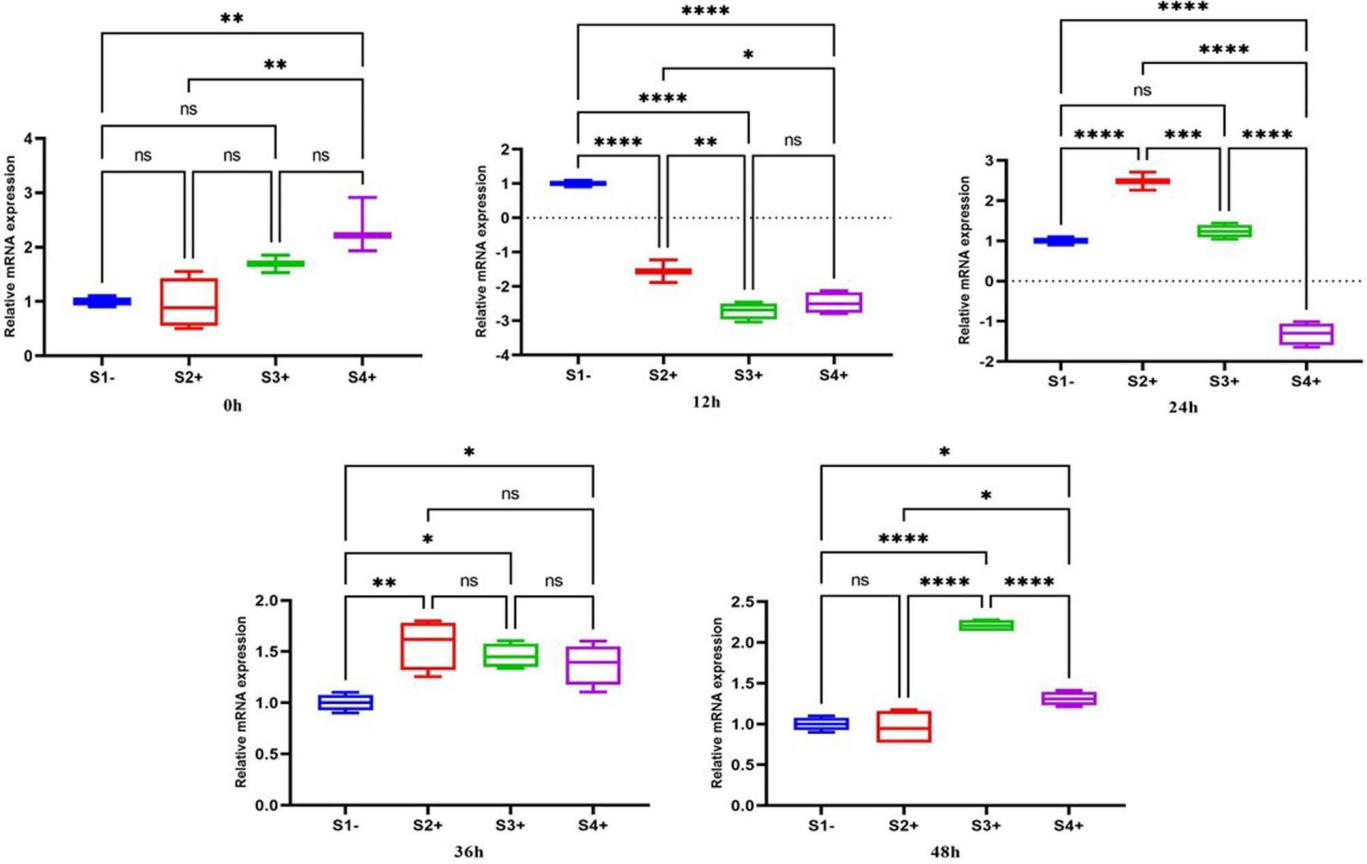
Gene expression of the *HSP83* gene in three LRV2 + isolates (S2+, S3+, and S4+) compared to LRV2- isolate (S1-) at different times after co-infection. Data analysis was done using two-way ANOVA for repeated measurements followed by the Tukey test. Bars represent mean ± SD. * Symbol represents the meaningful difference between groups. (*P ≤ 0.05, **P ≤ 0.01, ***P ≤ 0.001, ****P ≤ 0.0001)

The expression of *HSP83* gene revealed significant upregulation at zero for S4+ (2.3; *P*-value = 0.01) compared to the S1- (LRV-). At 12 h after co-infection, significant downregulation was observed in all isolates compared to S1-. The expression of *HSP83* gene after 24 h was upregulated in S2+ (2.5; *P*-value = 0.0001) and S3+, but was still downregulated in S4+ (-1.35; *P*-value = 0.0001). At 36 h, all isolates were upregulated, and at 48 h, except S2+, the expression of *HSP83* was significantly upregulated compared to S1- (Fig. 2).

#### MPI

**Fig. 3:**
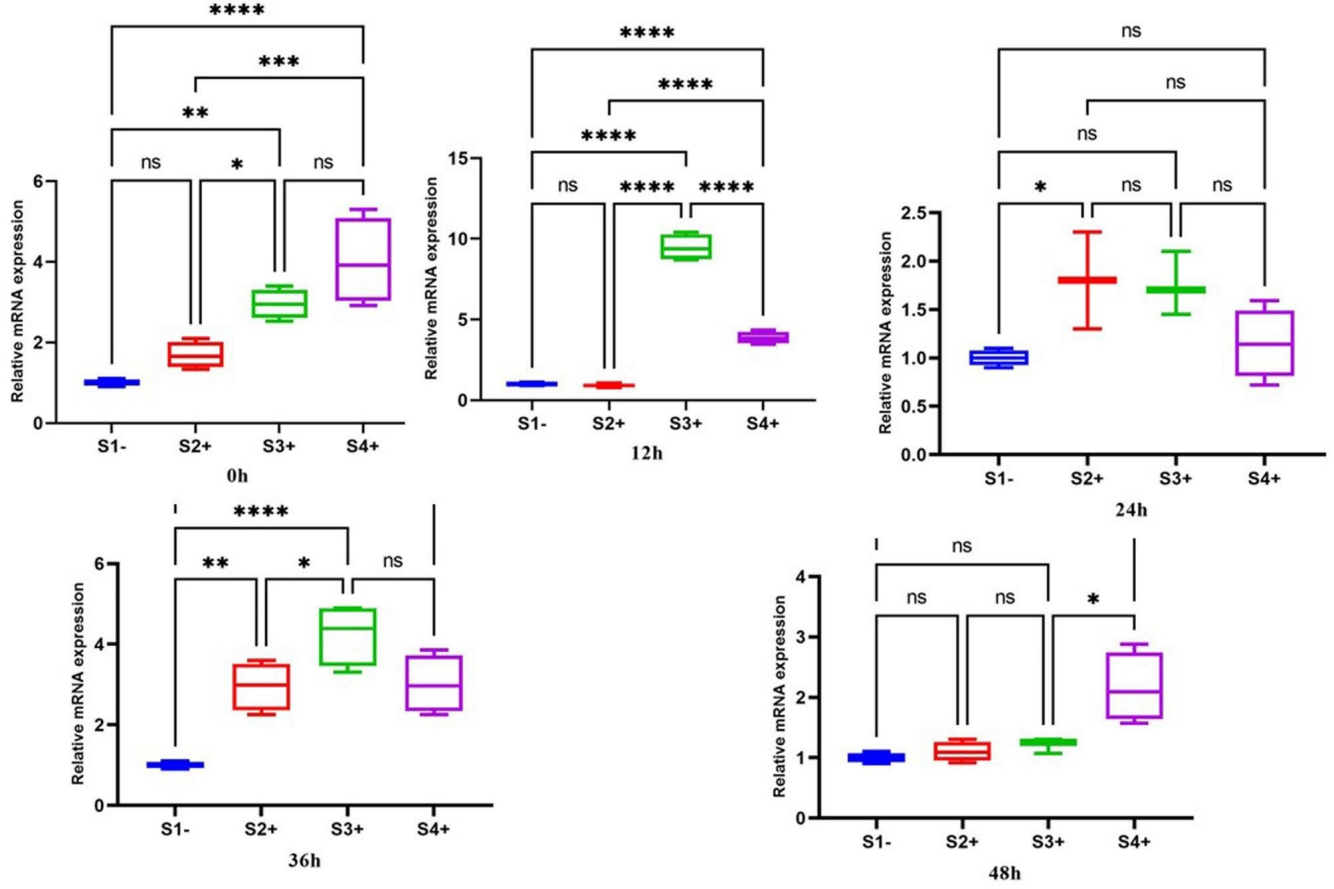
Gene expression of the *MPI* gene in three LRV2 + isolates (S2+, S3+, and S4+) compared to one LRV2- isolate (S1-) at different times after co-infection. Data analysis was done using two-way ANOVA for repeated measurements followed by the Tukey test. Bars represent mean ± SD. * Symbol represents the meaningful difference between groups. (*P ≤ 0.05, **P ≤ 0.01, ***P ≤ 0.001, ****P ≤ 0.0001)

The expression of the *MPI* gene was upregulated at zero time-point, with the highest expression in the S4 + isolate (4; *P*-value = 0.0001). At the 12 h time-point, significant upregulation was observed in the S3+ (9.4; *P*-value = 0.0001) and the S4+ (3.8; *P*-value = 0.0001) isolates. During the 24 h period after co-infection, an increase was observed in the S2+, S3+, and S4 + isolates. By the 36 h time-point, the expression of the *MPI* gene was significantly increased in all isolates. At 48 h, significant upregulation was observed in the S4 + isolate (2.1; *P*-value = 0.0176) (Fig. 3).

### Pro-inflammatory biomarker

#### NLRP3

**Fig. 4:**
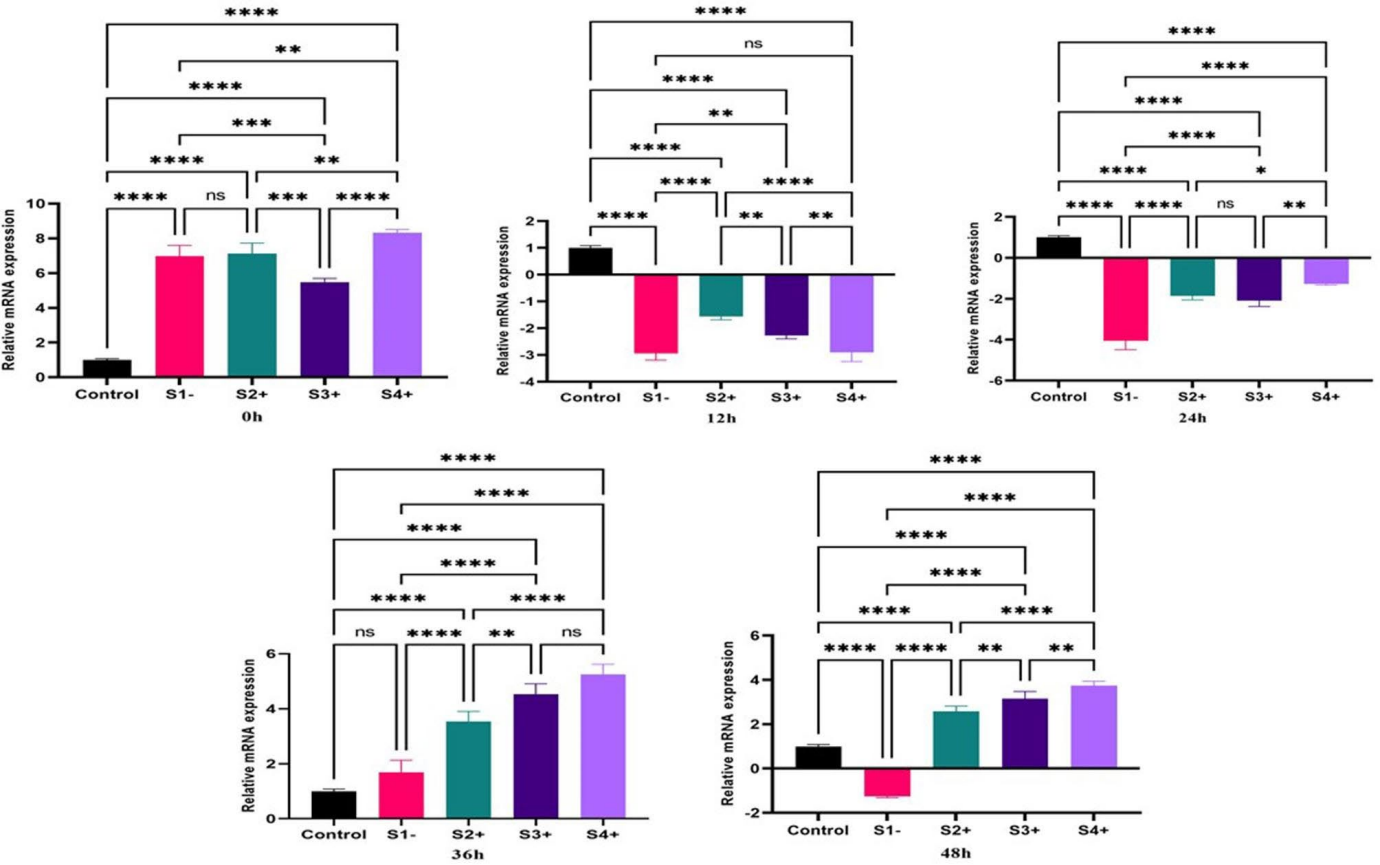
The expression of the *NLRP3* gene in three LRV2 + isolates (S2+, S3 + and S4+), and LRV2- isolate (S1-) compared to control (uninfected macrophage) at different times after co-infection. Data analysis was done using two-way ANOVA for repeated measurements followed by the Tukey test. Bars represent mean ± SD. * Symbol represents the meaningful difference between groups. (*P ≤ 0.05, **P ≤ 0.01, ***P ≤ 0.001, ****P ≤ 0.0001)

The expression of *NLRP3* gene revealed significant upregulation at zero time-point for S1- (6.98; *P*-value = 0.0001) and S2+ (7.12; *P*-value = 0.0001) S3+ (5.48; *P*-value = 0.0001) and S4+ (8.31; *P*-value = 0.0001) compared to the control (un-infected macrophage). At 12, and 24 h, all isolates were significantly downregulated compared to the control. At 36 and 48 h, all LRV2 + isolates were upregulated compared to the LRV2- isolate and control groups. (Fig. 4).

#### IL-18

**Fig. 5:**
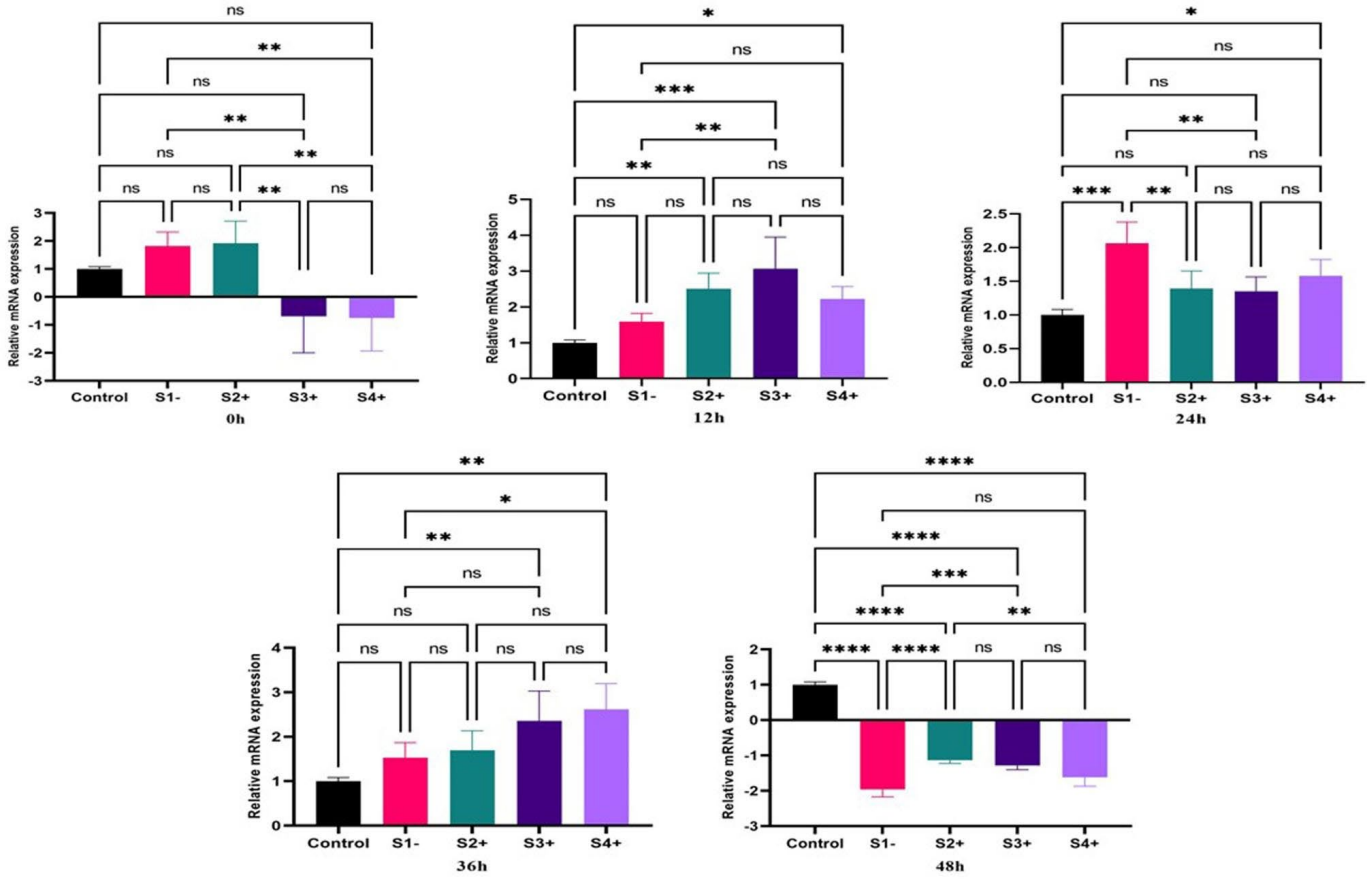
The expression of the *IL18* gene in three LRV2 + isolates (S2+, S3 + and S4+) and LRV2- isolate (S1-) compared to control (uninfected macrophage) at different times after co-infection. Data analysis was done using two-way ANOVA for repeated measurements followed by the Tukey test. Bars represent mean ± SD. * Symbol represents the meaningful difference between groups. (*P ≤ 0.05, **P ≤ 0.01, ***P ≤ 0.001, ****P ≤ 0.0001)

The results showed significant changes in the *IL-18* gene expression at the time of zero compared to the control. The S1- and S2 + were upregulated, but S3 + and S4 + were downregulated. An upregulation was observed at time-point 12, in S2+, S3+, and S4+ (2.51; *P*-value = 0.01), (3.06; *P*-value = 0.001), (2.22; *P*-value = 0.05), respectively. The expression of the *IL-18* gene during the time-points 24 and 36 h was upregulated, while the highest gene expressions of the *IL-18* gene was at 36 h and in S4+ (2.61; *P*-value = 0.01). At the 48 h time-point, all isolates were significantly downregulated compared to control (Fig. 5).

#### IL-1β

**Fig. 6:**
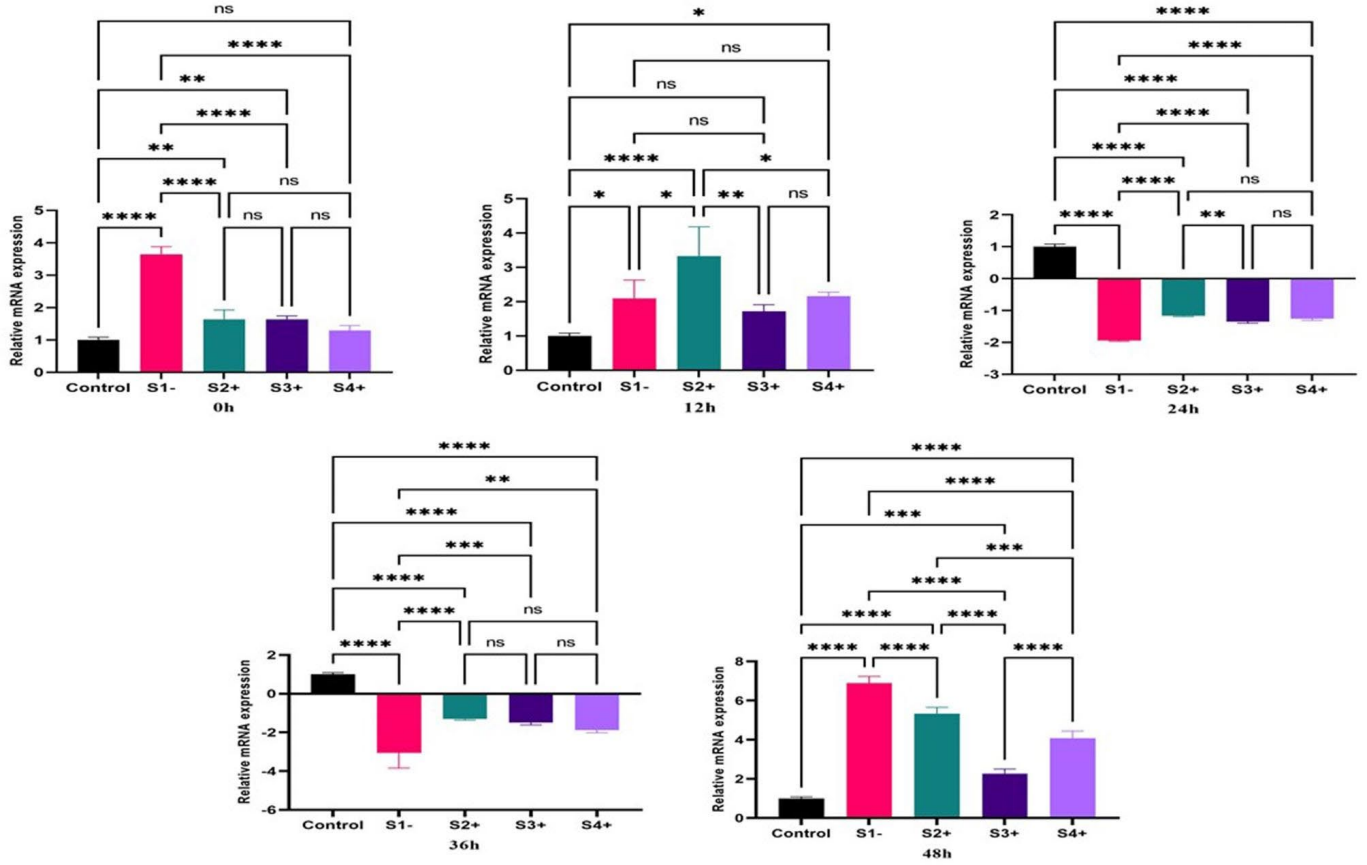
The expression of the *IL1β* gene in three LRV2 + isolates (S2+, S3 + and S4+), and LRV2- isolate (S1-) compared to control (uninfected macrophage) at different times after co-infection. Data analysis was done using two-way ANOVA for repeated measurements followed by the Tukey test. Bars represent mean ± SD. * Symbol represents the meaningful difference between groups. (*P ≤ 0.05, **P ≤ 0.01, ***P ≤ 0.001, ****P ≤ 0.0001)

The results of real-time PCR showed significant changes of the *IL-1β* gene at the time of zero compared to control. All isolates were significantly upregulated compared to control, but S1- revealed higher upregulation compared to LRV2 + isolates (3.6; *P*-value = 0.0001). At time-point 12 an upregulation was still observed in S1-, S2+, S3 + and S4+, but S2 + showed higher gene expression compared to other isolates (3.32; *P*-value = 0.0001). The expression of *IL-1β* gene during the time-points 24 and 36 h was significantly decreased in all isolates, but at 48 h, all isolates were significantly upregulated compared to other time point (Fig. 6).

## Discussion

The pathogenicity of *Leishmania* parasites is influenced by multiple factors, most importantly including *Leishmania* species, VFs expression, and the host’s immune responses [15, 16]. Recent findings have indicated that LRVs may intensify the severity of the disease, boost invasion of *Leishmania* parasite, and modulate immune responses [15, 17, 39, 40]. These findings suggest an association between LRVs and the clinical outcomes of leishmaniasis. The role of LRV1 in the pathogenesis of New World *Leishmania* species has been investigated [15, 16, 41]. However, there is limited data regarding the correlation between LRV2 and Old World *Leishmania* species [17]. Therefore, in this study, we evaluated the impact of LRV2 on the expression of VFs and pro-inflammatory biomarkers in response to *L*. *major* isolates, both LRV2 + and LRV2-, at different time points after co-infection.

GP63 is known to play a critical role in the attachment and entry of *Leishmania* promastigotes into macrophages. GP63 participates in other important processes, such as modulation of the host immune responses, degradation of host cell components, and further contributing to the pathogenesis of *Leishmania* infections [42, 43]. An in vivo study demonstrated that GP63-deficient *L*. *major* significantly reduces the development of CL lesions in mice, suggesting that GP63 does not significantly influence the pathogen-induced inflammatory cell recruitment, but may affect inflammatory cell activation and functions [43]. However, conflicting results have been released about the role of LRV in the expression of GP63. Kariyawasam et al. [16]. reported no significant difference in *GP63* gene expression between LRV1 + and LRV1- groups. Our data revealed an increasing trend in the expression of *GP63* in LRV2 + isolates compared to LRV2-. It is noteworthy that our findings on the expression of *GP63* are closely aligned with the results reported by Rahmanipour et al. [17]. Therefore, it seems that the *GP63* gene represents higher expression in LRV2 + isolates compared to LRV2-. Nevertheless, further studies are required to validate and confirm these results.

The expression of *HSP83* gene in *Leishmania-*infected macrophages is upregulated. This upregulation plays a significant role in both parasite survival and replication [21]. It was shown that a higher concentration of HSP83 is associated with active mucosal and cutaneous ulcers, suggesting a positive correlation between *HSP83* and the pathogenicity of *Leishmania* species [44, 45]. In this study, the expression of *HSP83* gene showed an increase at all time-points in LRV2 + compared to LRV- isolates, although a downregulation was observed at 12 h in LRV2 + isolates. However, the outcome of the presence of LRVs on the expression of HSP-related genes is controversial. For example, Rahmanipour et al. [17]. observed higher levels of *HSP70* gene expression in the initial hours for the LRV2 + strain, while it was downregulated at the final time-points. In contrast, Kariyawasm et al., [16] reported higher expression of *HSP90* in LRV- strains compared to LRV1 + strains. Therefore, it seems that the role of species or strains of *Leishmania* and the presence of LRV may affect the expression of HSPs. Generally, HSP83 is thought to be constitutively expressed, which is consistent with our findings [16].

MPI is involved in the recruitment of other VFs including lipophosphoglycan (LPG) and GP63, and the lack of this protein has been associated with slow growth in *Leishmania* parasites [27]. Kariyawasm et al., [16] reported higher expression of *MPI* gene in LRV- strains compared to LRV1 + strains. In contrast, current findings have shown that the expression *MPI* gene was increased in all isolates of LRV2 + compared to LRV2-. Therefore, it may be concluded that LRV2 plays an important role in the upregulation of VFs genes. However, to validate this observation, further investigations are required. For this purpose, monitoring of the ulcer progression, response to treatment, and clinical presentation of CL lesions should be considered.

Different results have been reported regarding the role of cytokines and inflammasomes in the pathogenesis of CL [15, 34, 41, 46]. As an early response to *Leishmania*, activation of inflammasomes, particularly NLRP3, is a vital part of the immune response to the parasite. Upon stimulating NLRP3, caspase-1 is activated through auto-proteolysis, leading to the activation of pro-IL-18 and pro-IL-1β [47, 48]. Ives et al., [26] suggested that LRV may directly activate inflammatory signaling in macrophages, which leads to the activation of cytokines and chemokines. Therefore, LRV is a potential ligand for the activation of toll-like receptor (TLR) 3 and subsequent activation of the NLRP3 inflammasome [26]. Our results demonstrated that there was no difference in NLRP3 expression between LRV2 + and LRV2- isolates, during the initial hours, but at 48 h, LRV2 + isolates significantly increased the expression levels of *NLRP3* gene compared to LRV2-. de Carvalho et al. [40]. reported an inverse association between inflammasome activation and the severity of leishmaniasis, supporting a protective role of the inflammasome during *Leishmania* infection. Therefore, it can be concluded that the presence of LRV1 dampens NLRP3 activation to favor infection and pathogenesis of *Leishmania* parasite. Nevertheless, the activation of the NLRP3 plays a crucial role in determining the outcome of leishmaniasis [34]. Hartley et al., [49] reported no significant difference in NLRP3 expression between LRV1 + and LRV1- in *L*. *guyanensis.* Therefore, they demonstrated that *L*. *guyanensis* evades inflammasome activation, regardless of the presence of LRV1. Indeed, there is limited understanding of the signaling pathways that trigger NLRP3 activation in response to *Leishmania* infection. Further research is needed to elucidate the specific mechanisms through which *Leishmania* parasites induce NLRP3 activation and the subsequent inflammatory responses [39].

The role of IL-1β and IL-18 in *Leishmania* infection has been the subject of numerous studies [39, 46, 48]. It was reported that IL-1β can modulate the immune responses, while IL-18 shifts the T-cell activation pathway towards Th2, however, both cytokines contribute to the progression of the disease [50, 51]. Notably, IL-1β has been identified as a significant signaling factor for host resistance against infection, as this cytokine transmits signals through IL-1R and myeloid differentiation primary response protein (MyD) 88, leading to the induction of NOS2-mediated nitric oxide (NO) production. In addition, it was suggested that IL-1β plays a role in increasing NO production, leading to reduced parasite proliferation and enhanced resistance to *Leishmania* infection. In our study, the expression of *IL-1β* gene was higher in the LRV- isolate than the LRV2 + isolates at the initial and final hours. In the line of our results, Kariyawasam et al., [15] and Carvalho et al., [40] reported similar findings, reporting higher expression of *IL-1β* gene in LRV1- compared to LRV1+. Hence, it appears that the presence of LRV plays a significant role in suppressing the activity of the immune system and the expression of pro-inflammatory cytokines. The expression of IL-1β in LRV2 + isolates was reported higher than in LRV- isolate during the early hours, but in contrast to our findings, the expression of *IL-1β* gene was lower in LRV- isolate compared to LRV2 + isolate at the final hours [17].

IL-18 is a pro-inflammatory cytokine that plays a protective role against pathogenesis factors of the *Leishmania* parasite and contributes to innate and adaptive immunity. Evidence suggests that IL-18 plays a critical role in modulating T cell responses during *L*. *major* infection [51–53]. Some studies indicate a positive role for IL-18 in promoting Th1 responses and resistance against *Leishmania* species infection, while conflicting results showed that IL-18 may enhance Th2-biased responses and causes susceptibility to the parasites [51, 52]. It was suggested that IL-18 may induce development of Th1 and natural killer (NK) cells and production of IFNγ via overexpression of IL-18R on Th1 and NK cells [54, 55]. In addition, IL-18 induces an IFNγ-independent immunity against *Leishmania* parasites [56]. In contrast, IL-18 seems to produce and release Th2 cytokines like IL-4 and IL-13 [57–59], which are protective against *L. donovani*, while induces susceptibility to *L. major* [58]. However, the role of IL-18 in *Leishmania* infections remains vague and depends to the *Leishmania* species and host genetics. In our study, we observed downregulation of *IL-18* gene in the LRV2 + isolates compared to the LRV2- isolate in the early and middle hours, however, there was an upregulation during the final hour. However, it is necessary to fully elucidate the mechanisms behind the activation of the host’s immune responses in leishmaniasis, led by IL-18.

## Conclusion

Our observations indicate that the presence of LRV2 + in *L*. *major* in comparison to LRV2- leads to an increase in the expression of VFs (*GP63*, *HSP83*, and *MPI* genes), while there is a declining trend in the expression of pro-inflammatory biomarkers (*NLRP3*, *IL-18*, and *IL-1β* genes). However, it is crucial to take into account the influence of various factors, including the host immune response, different *Leishmania* strains, the presence of VFs, and the expression of cytokines, in addition to the LRV status.

Collectively, the pathogenesis of *Leishmania* parasites is highly complex, particularly when attempting to establish a link between the pathogenesis and *Leishmania* viruses. Understanding the interplay between the parasite, the virus, and the host immune responses is a critical challenge, and further investigations and comprehensive studies are required to unravel the intricate mechanisms involved in the pathogenesis of *Leishmania* parasites and the potential influence of LRVs.

### Electronic supplementary material

Below is the link to the electronic supplementary material.

**Supplementary Material 1:** Suppl Fig 1. The 1.5% gel electrophoresis of extracted RNA from isolated L. major. The presence of three 5s, 15s, and 28s confirmed the extraction of RNA from L. major sample.

**Supplementary Material 2:** Suppl fig 2. The gel electrophoresis of conventional PCR for detection of Leishmania spp. Line 1: ladder 100 bp, Line 2: negative control; Line 3: positive control for L. tropica; Line 4: positive control for L. major, Line 5–8: samples.

## Data Availability

The datasets generated and analyzed during the current study are available from the corresponding author on reasonable request.
