# Supplementary material for: The effects of Leishmania RNA virus 2 (LRV2) on the virulence factors of L. major and pro-inflammatory biomarkers: an in vitro study on human monocyte cell line (THP-1)
Source: BMC Microbiol. 2023 Dec 14;23:398. doi: 10.1186/s12866-023-03140-0 (PMC10720061; doi:10.1186/s12866-023-03140-0)
Supplement: Supplementary file 1 — Supplementary Material 1: Suppl Fig 1. The 1.5% gel electrophoresis of extracted RNA from isolated L. major. The presence of three 5s, 15s, and 28s confirmed the extraction of RNA from L. major sample. [file 12866_2023_3140_MOESM1_ESM.pdf]

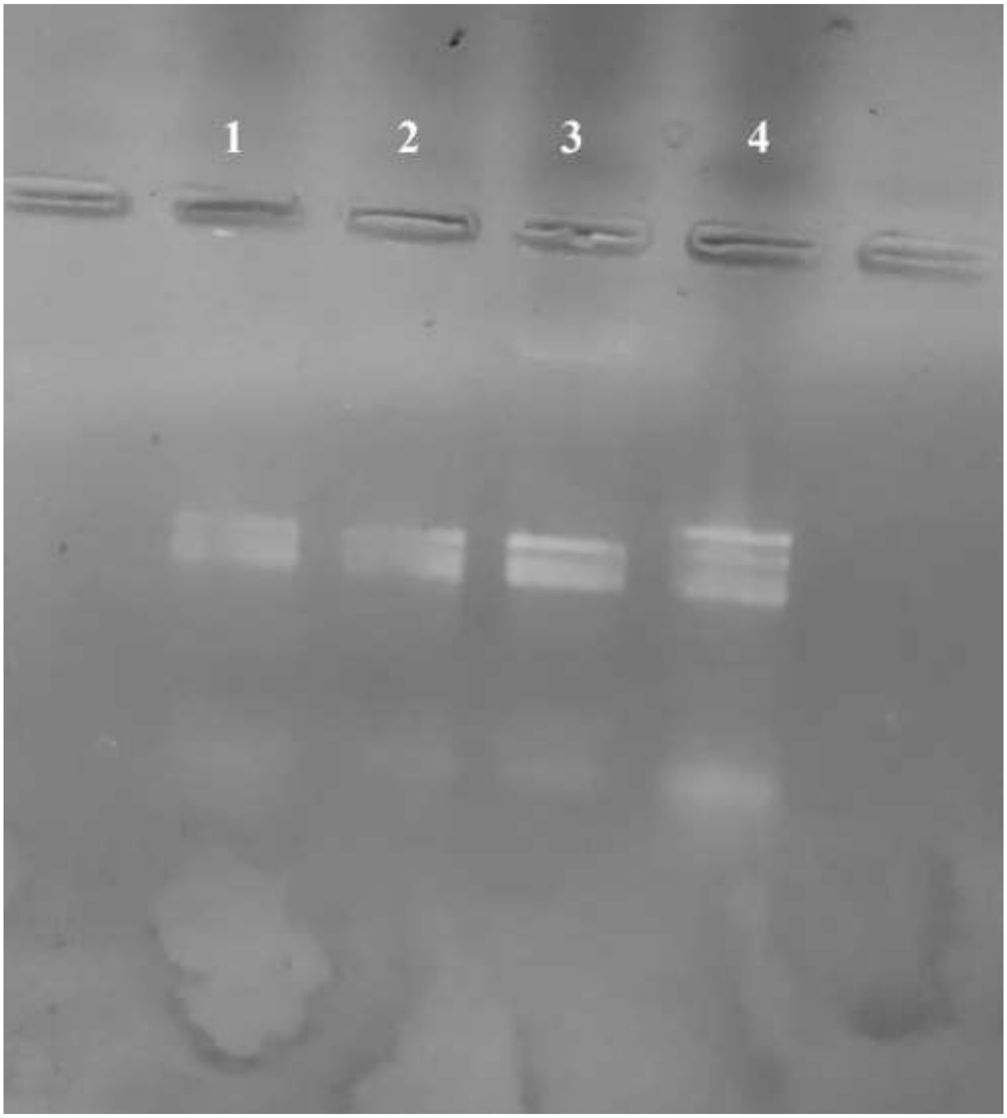

Suppl Fig 1. The 1.5% gel electrophoresis of extracted RNA from isolated *L. major*. The presence of three 5s, 15s, and 28s confirmed the extraction of RNA from *L. major* sample.
