# Supplementary material for: The effects of Leishmania RNA virus 2 (LRV2) on the virulence factors of L. major and pro-inflammatory biomarkers: an in vitro study on human monocyte cell line (THP-1)
Source: BMC Microbiol. 2023 Dec 14;23:398. doi: 10.1186/s12866-023-03140-0 (PMC10720061; doi:10.1186/s12866-023-03140-0)
Supplement: Supplementary file 2 — Supplementary Material 2: Suppl fig 2. The gel electrophoresis of conventional PCR for detection of Leishmania spp. Line 1: ladder 100 bp, Line 2: negative control; Line 3: positive control for L. tropica; Line 4: positive control for L. major, Line 5–8: samples. [file 12866_2023_3140_MOESM2_ESM.pdf]

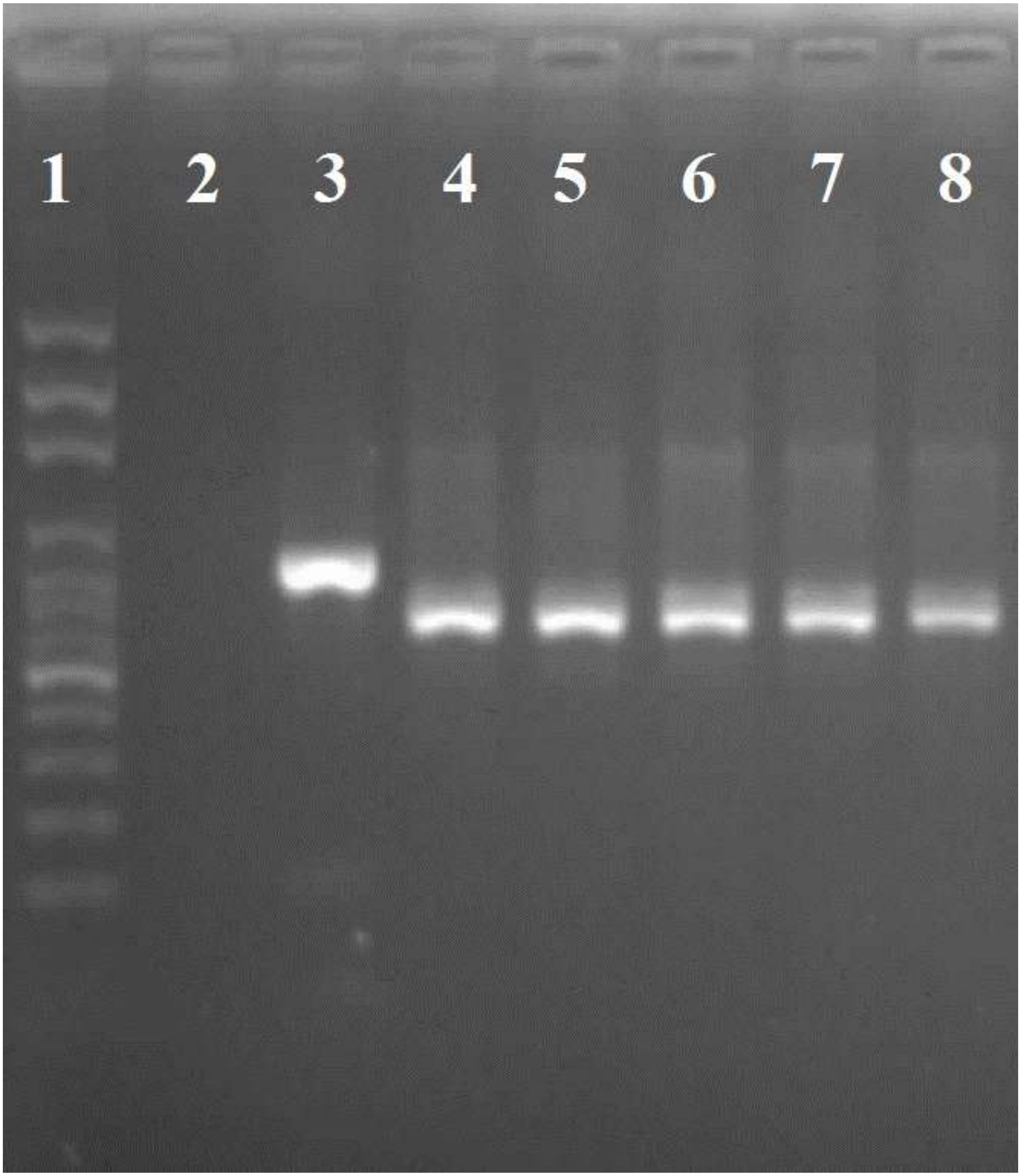

Suppl fig 2. The gel electrophoresis of conventional PCR for detection of *Leishmania* spp. Line 1: ladder 100 bp, Line 2: negative control; Line 3: positive control for *L. tropica*; Line 4: positive control for *L. major*, Line 5-8: samples.
